# Supplementary material for: Organ geometry channels reproductive cell fate in the Arabidopsis ovule primordium
Source: eLife. 2021 May 7;10:e66031. doi: 10.7554/eLife.66031 (PMC8219382; doi:10.7554/eLife.66031)

Supplemental Dataset 1- Gallery of wild-type (Col_0) ovules used for the 3D digital atlas related to Figure 1

Number of ovules segmented for the analysis: 92 *from 84 images*

Stage 0-I	n=21
Stage 0-II	n=17
Stage 0-III	n=11
Stage 1-I	n=15
Stage 1-II	n=11
Stage 2-I	n=10
Stage 2-II	n=7

Stage 0-I (1/2)

EM_C_25

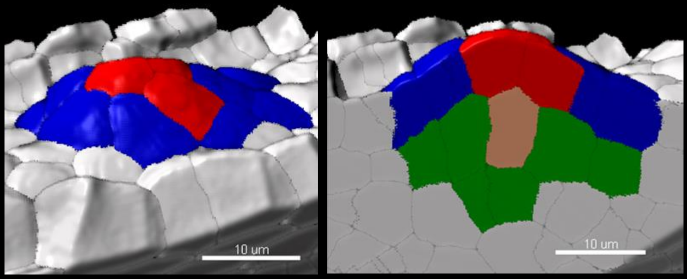

EM_C_58

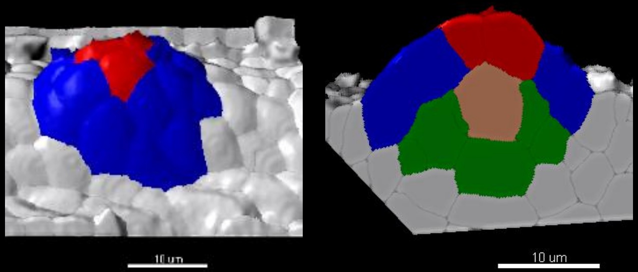

EM_C_59

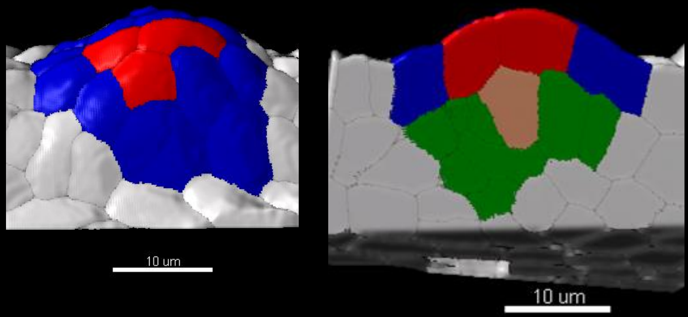

EM_C_60

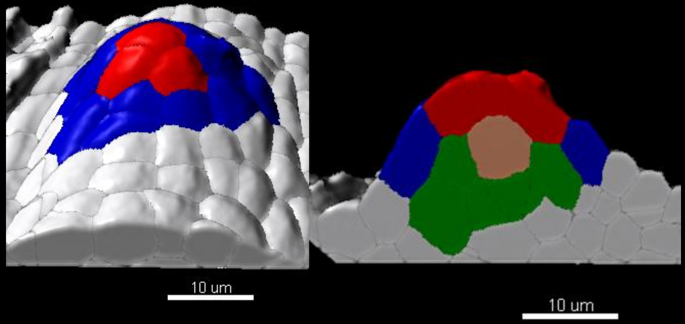

EM_C_76

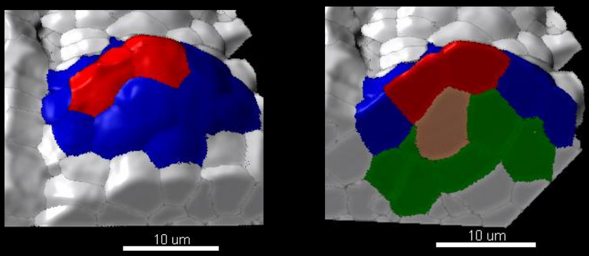

EM_C_149

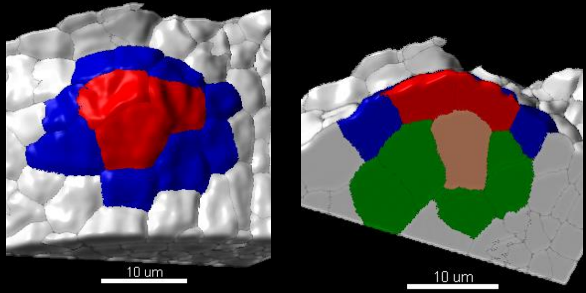

EM_C_237

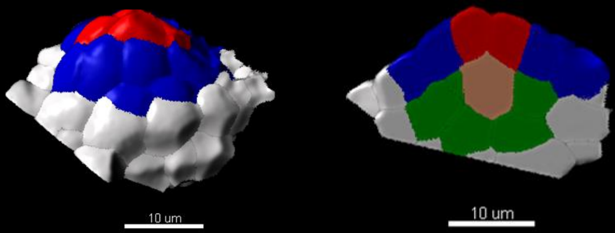

EM_C_240

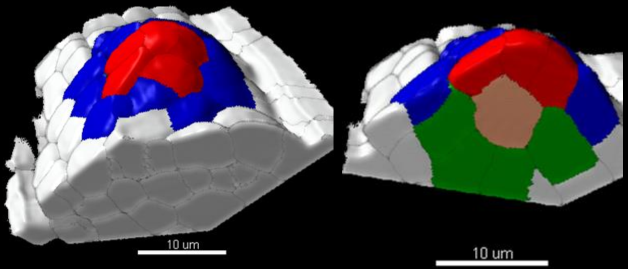

EM_C_241

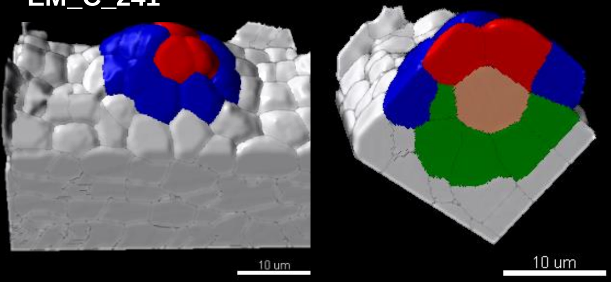

EM_C_242A

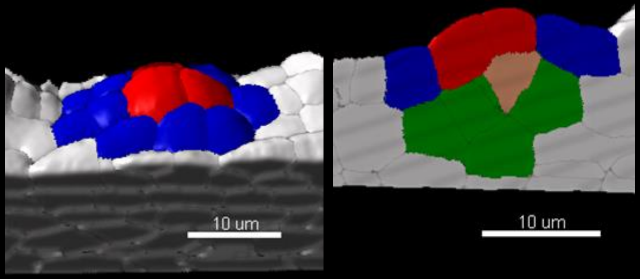

Stage 0-I (2/2)

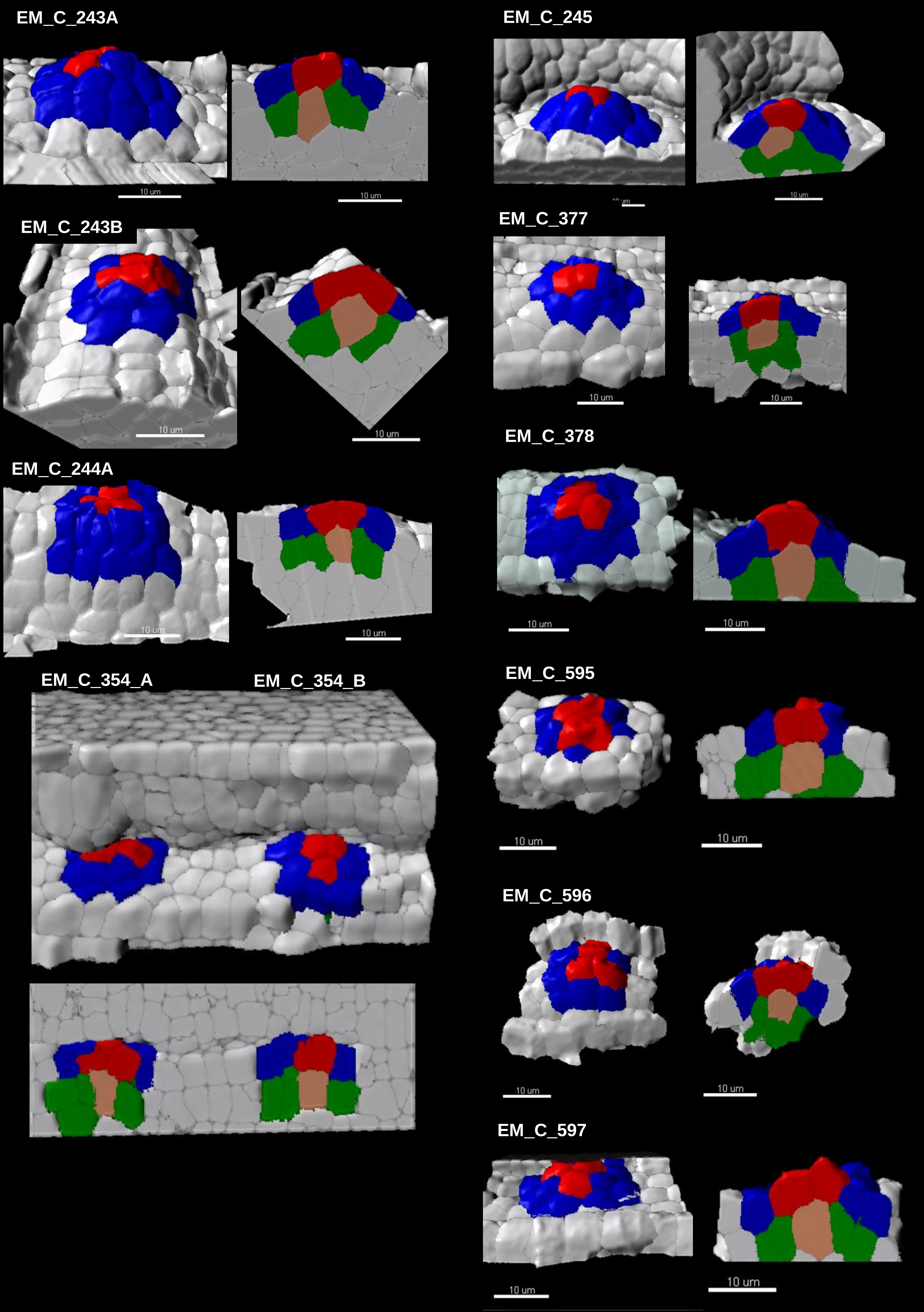
EM_C_243A
10 um
10 um
EM_C_245
10 um
EM_C_243B
10 um
10 um
EM_C_377
10 um
10 um
EM_C_378
10 um
10 um
EM_C_244A
10 um
10 um
EM_C_354_A
EM_C_354_B
EM_C_595
10 um
10 um
EM_C_596
10 um
10 um
EM_C_597
10 um
10 um

Stage 0-II (1/2)

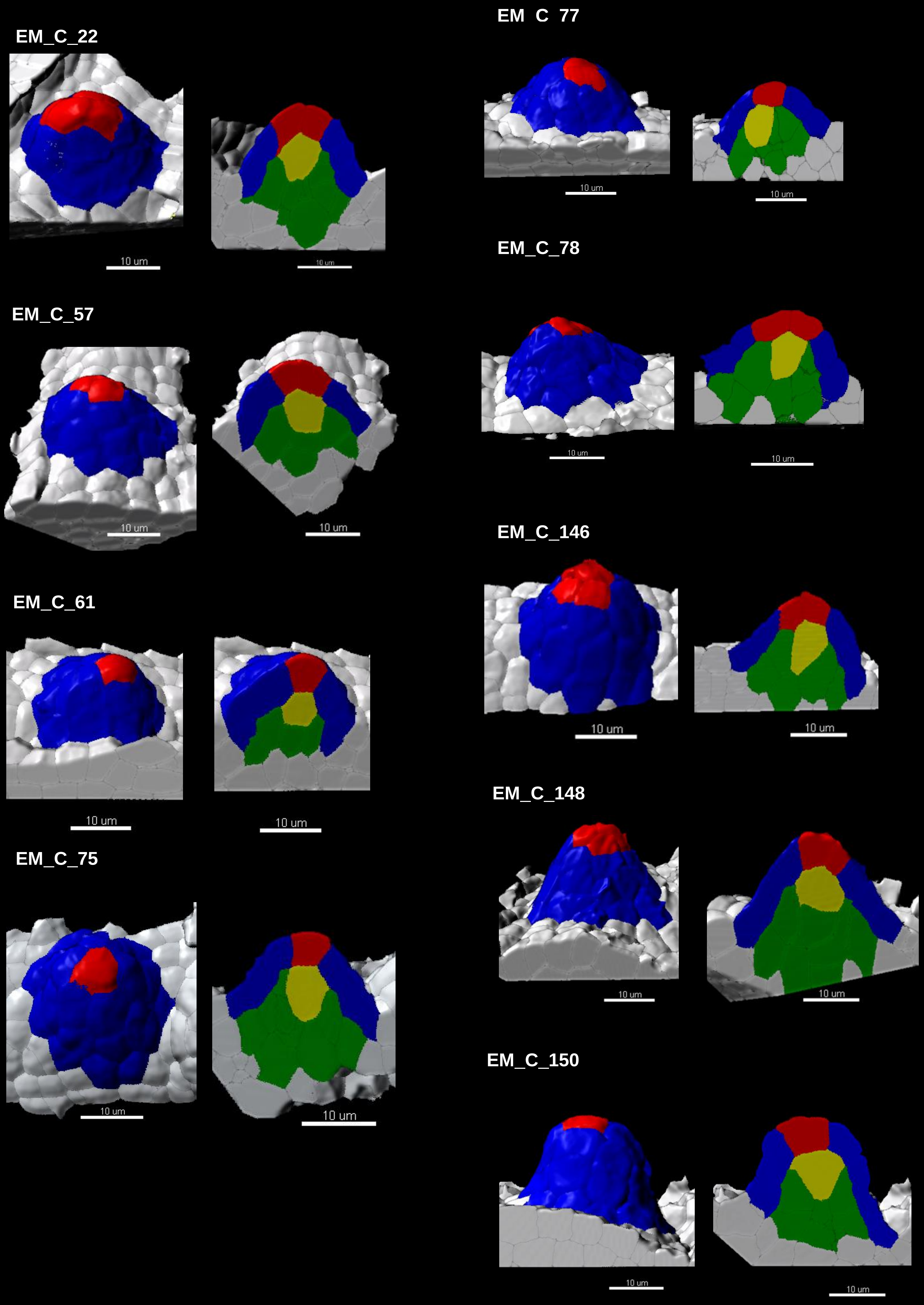
EM_C_22
10 um
10 um
EM_C_77
10 um
10 um
EM_C_57
10 um
10 um
EM_C_78
10 um
10 um
EM_C_61
10 um
10 um
EM_C_146
10 um
10 um
EM_C_75
10 um
10 um
EM_C_148
10 um
10 um
EM_C_150
10 um
10 um

Stage 0-II (2/2)

EM_C_211

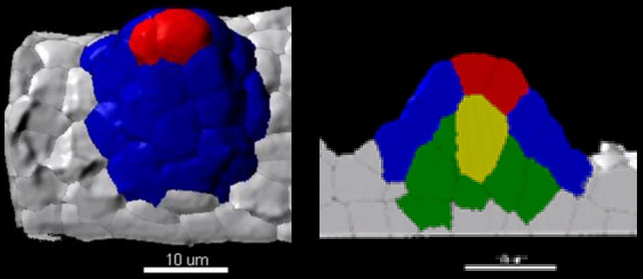

EM_C_212A

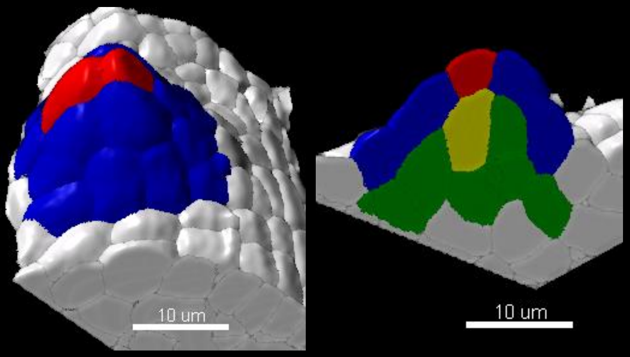

EM_C_212B

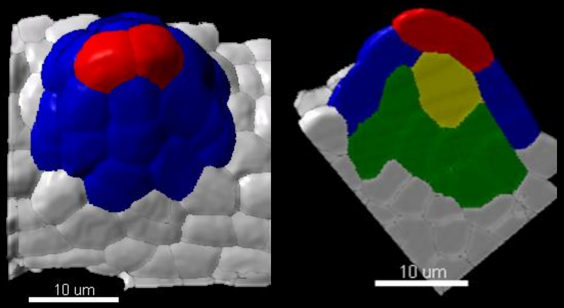

EM_C_213

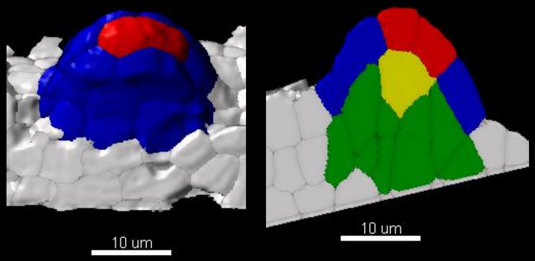

EM_C_214

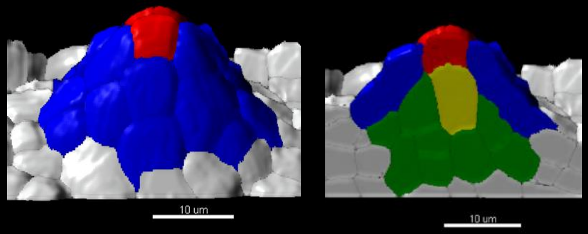

EM_C_238

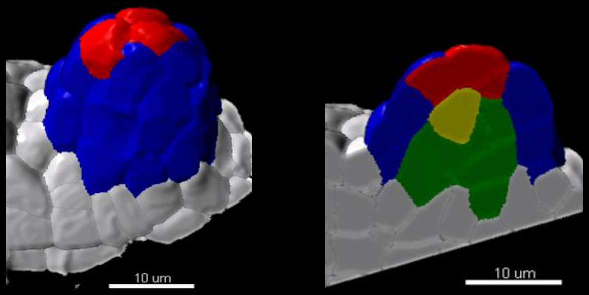

EM_C_239

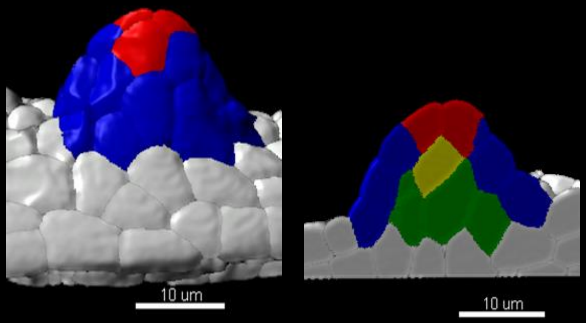

EM_C_246

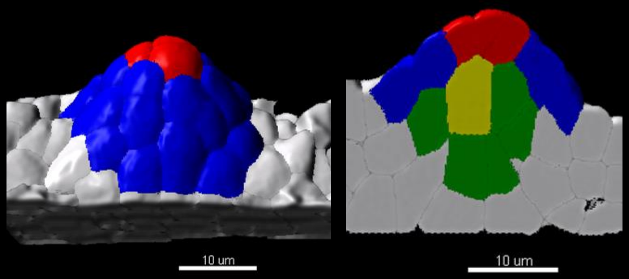

Stage 0-III

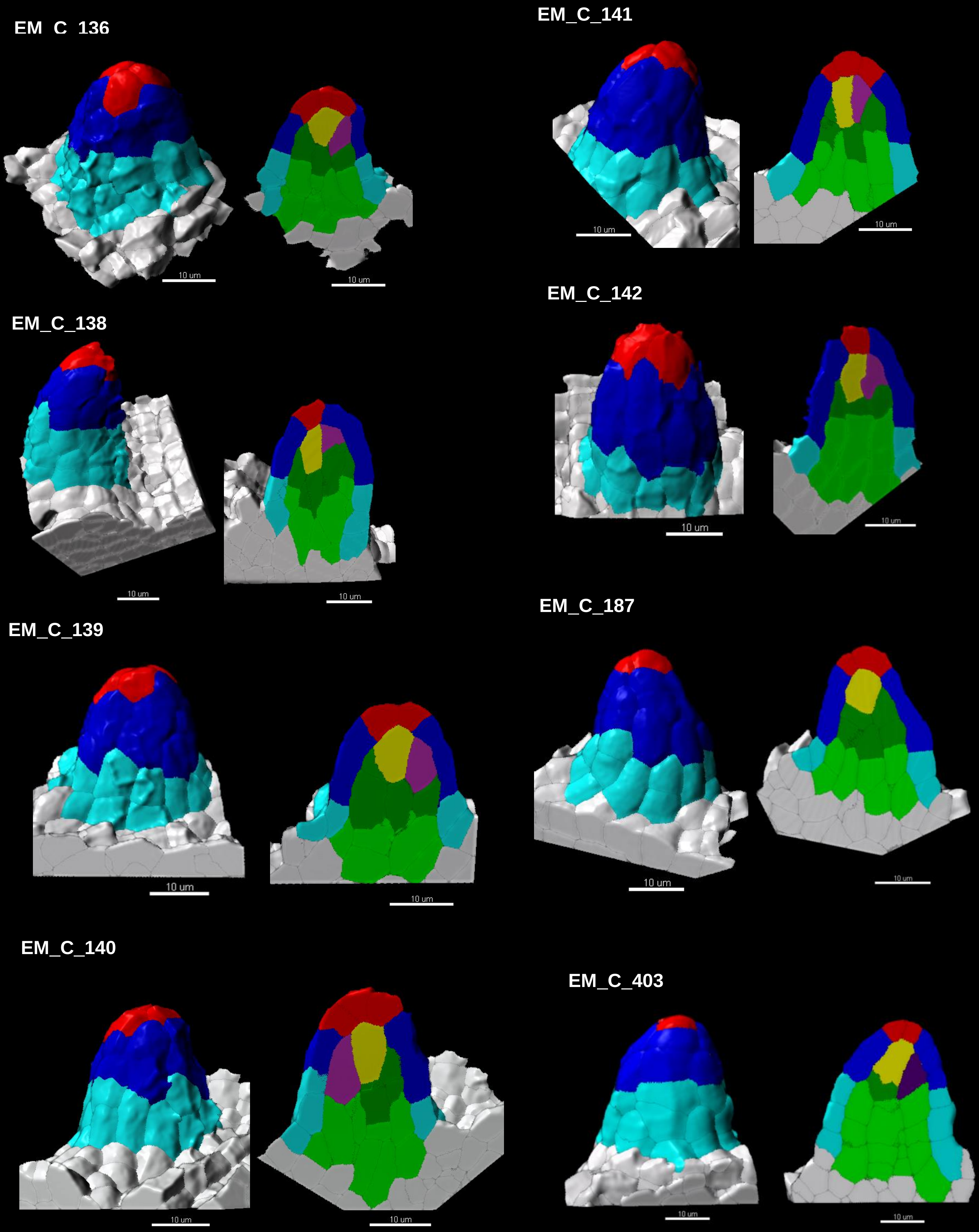
EM_C_136
EM_C_141
EM_C_142
EM_C_138
EM_C_187
EM_C_139
EM_C_140
EM_C_403
10 um

Stage 1-I (1/2)

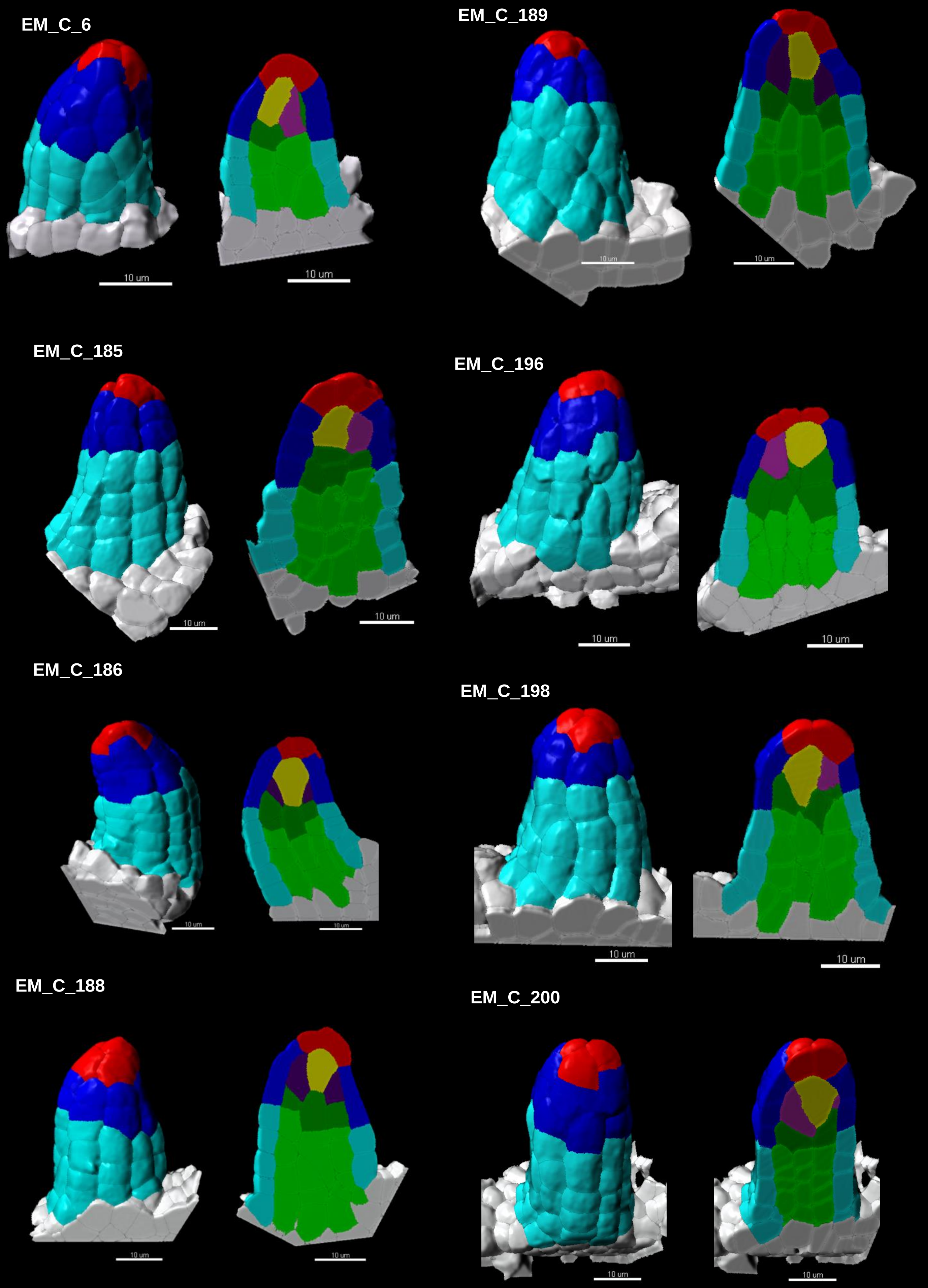
EM_C_6
10 um
10 um
EM_C_189
10 um
10 um
EM_C_185
10 um
10 um
EM_C_196
10 um
10 um
EM_C_186
10 um
10 um
EM_C_198
10 um
10 um
EM_C_188
10 um
10 um
EM_C_200
10 um
10 um

Stage 1-I (2/2)

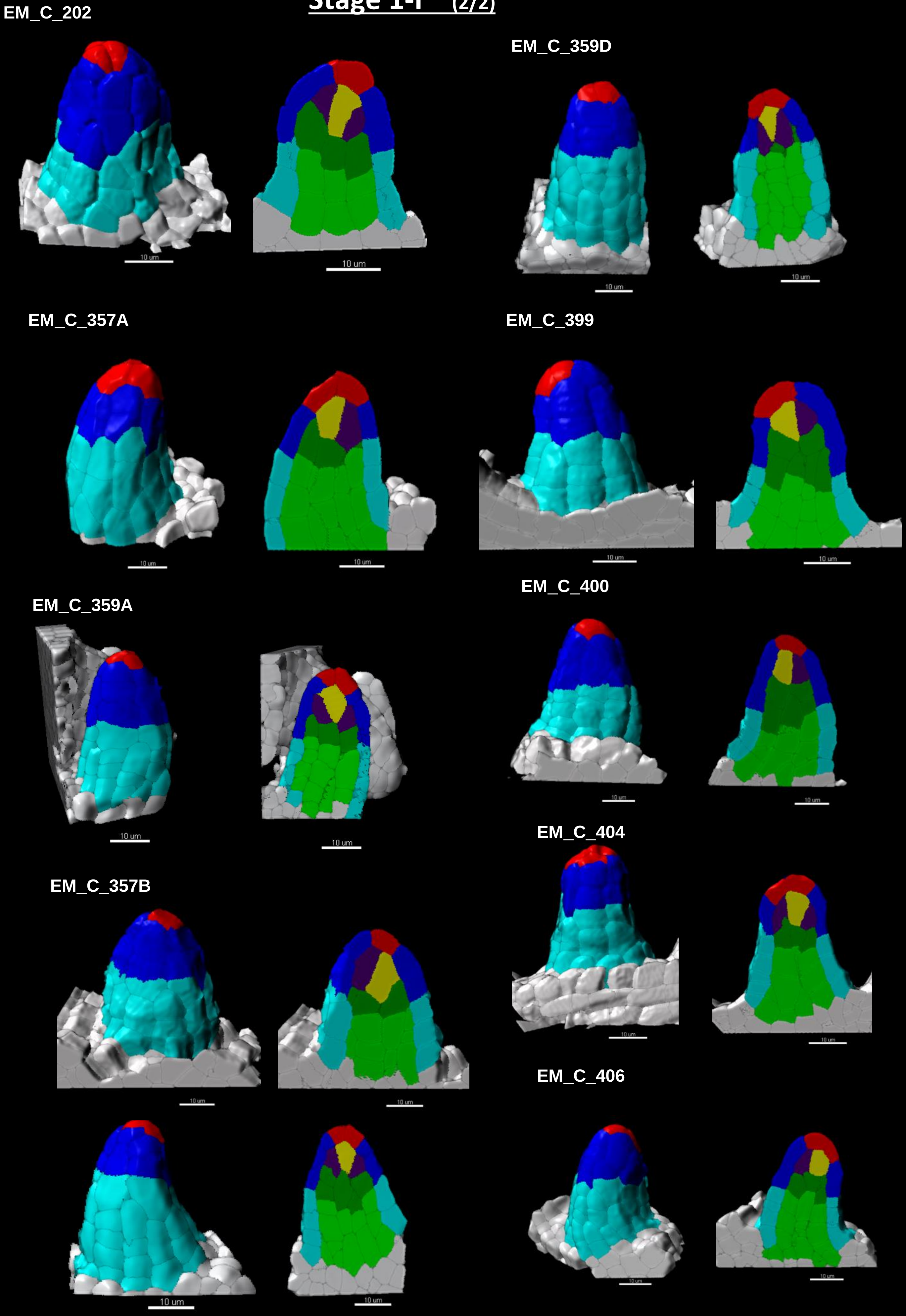
EM_C_202
10 um
10 um
EM_C_359D
10 um
10 um
EM_C_357A
10 um
10 um
EM_C_399
10 um
10 um
EM_C_359A
10 um
10 um
EM_C_400
10 um
10 um
EM_C_404
10 um
10 um
EM_C_357B
10 um
10 um
EM_C_406
10 um
10 um
10 um
10 um
10 um
10 um

Stage 1-II (1/2)

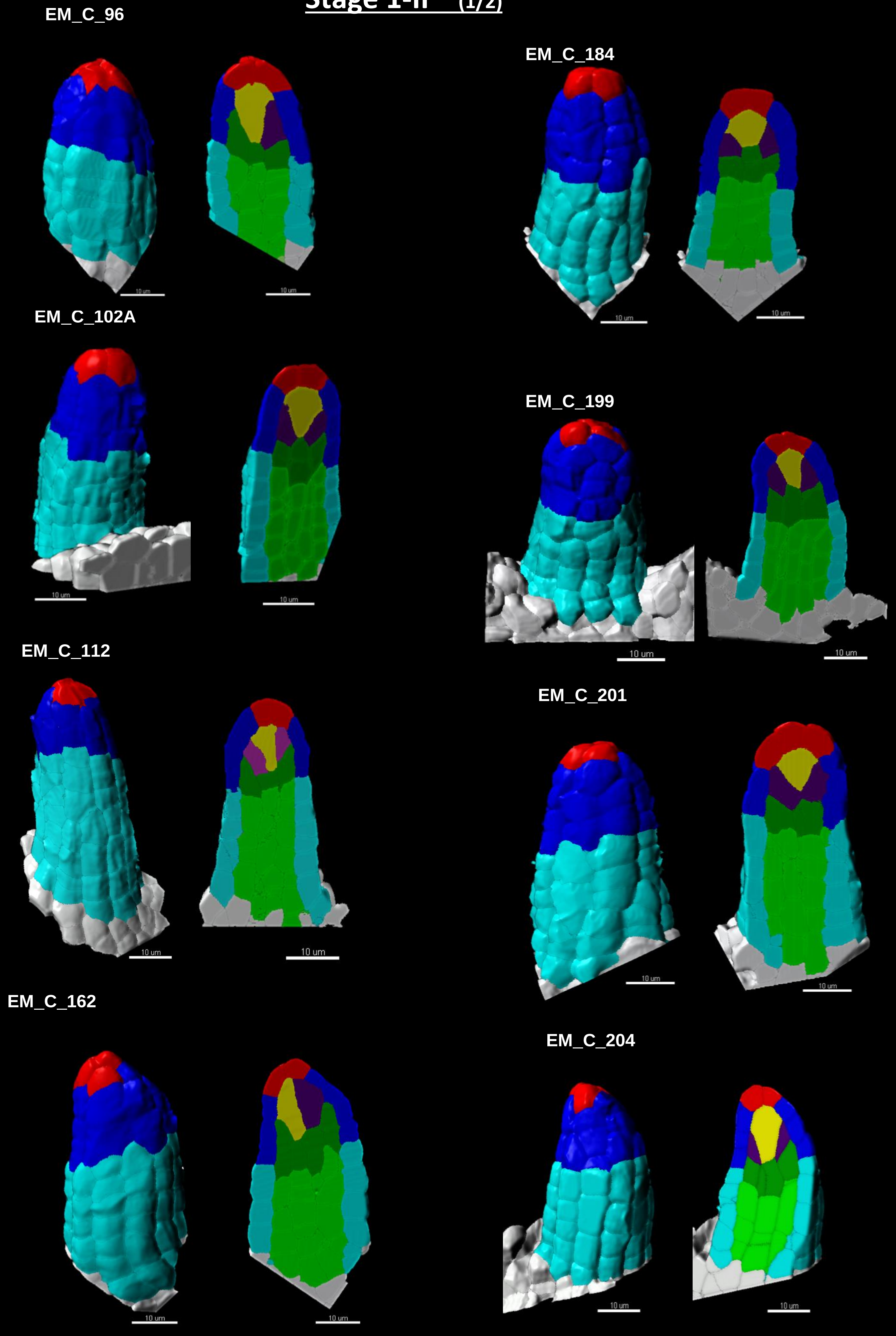
EM_C_96
10 um
10 um
EM_C_184
10 um
10 um
EM_C_102A
10 um
10 um
EM_C_199
10 um
10 um
EM_C_112
10 um
10 um
EM_C_201
10 um
10 um
EM_C_162
10 um
10 um
EM_C_204
10 um
10 um

Stage 1-II (2/2)

EM_C_359B

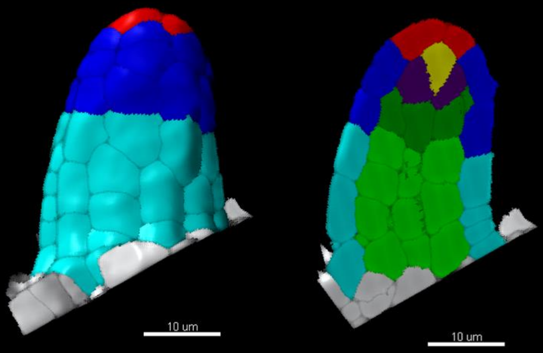

EM_C_607

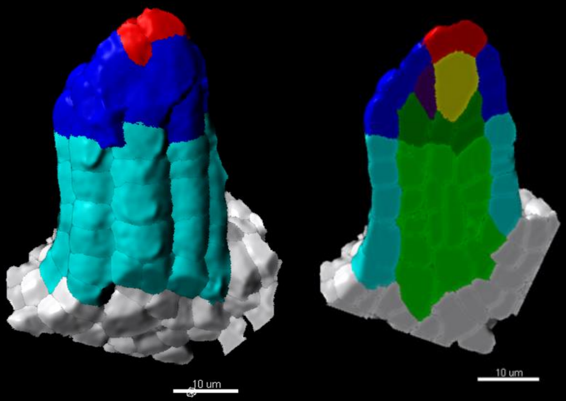

EM_C_160

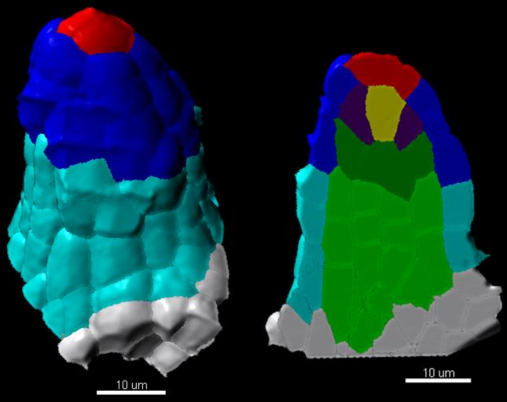

Stage 2-I

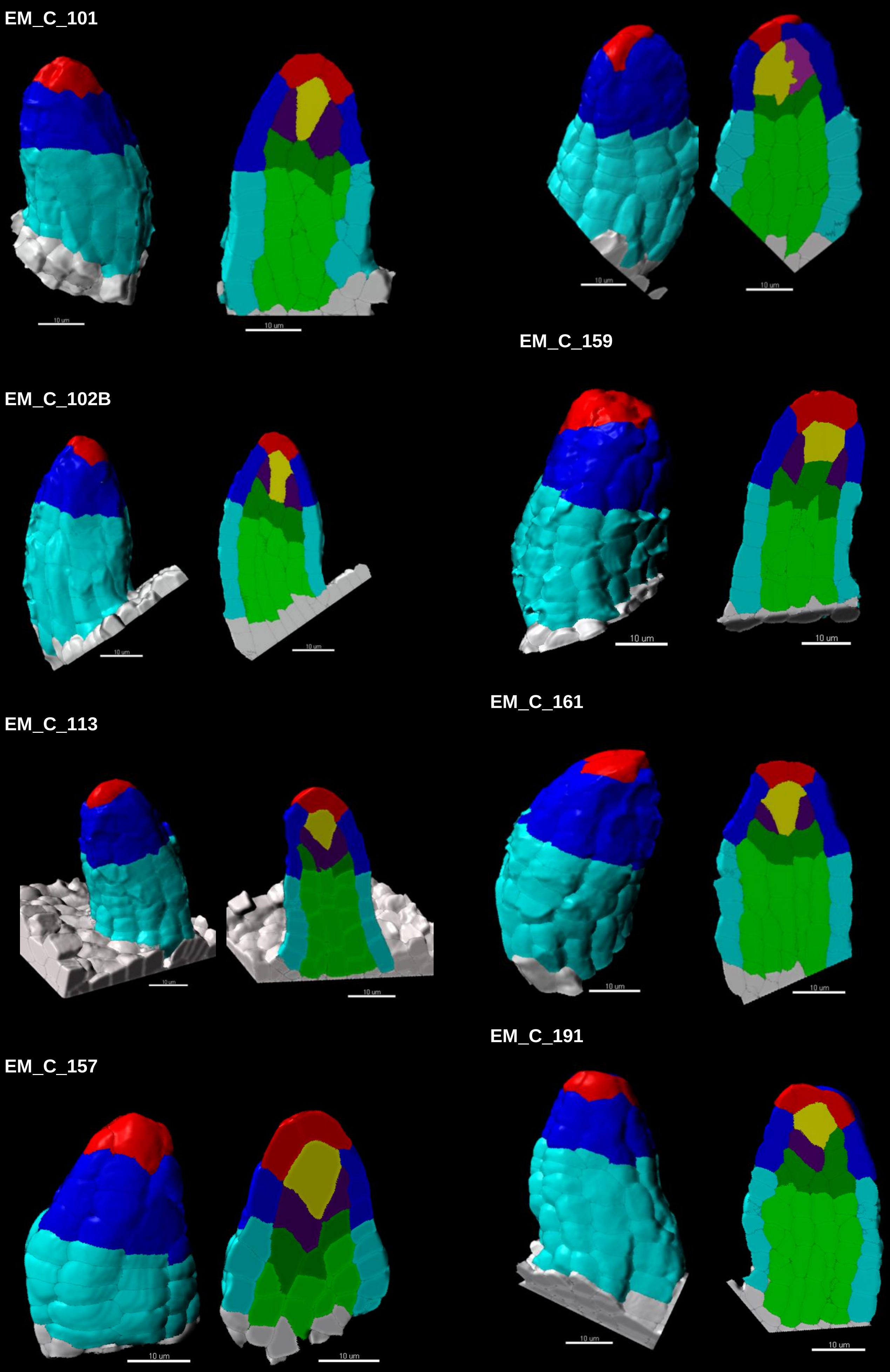
EM_C_101
EM_C_130
EM_C_159
EM_C_102B
EM_C_113
EM_C_161
EM_C_157
EM_C_191
10 um

Stage 2-I

EM_C_229

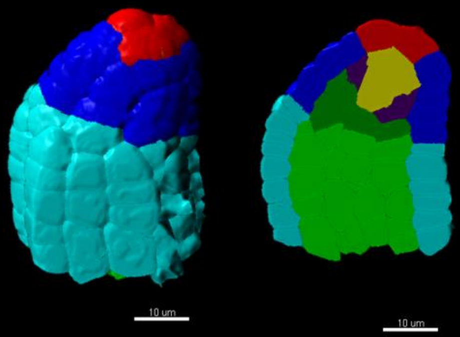

EM_C_232

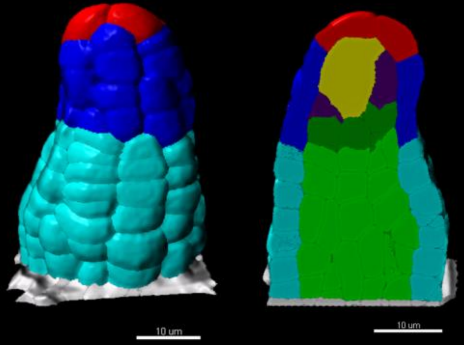

Stage 2-II

EM_C_81

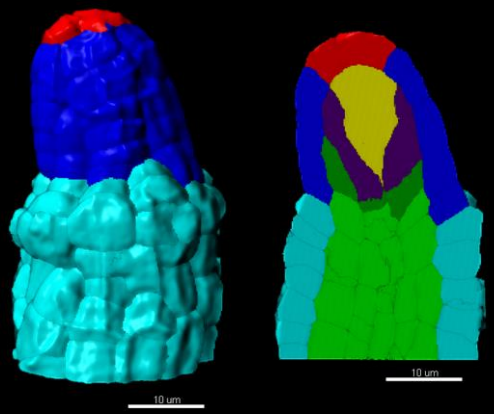

EM_C_128

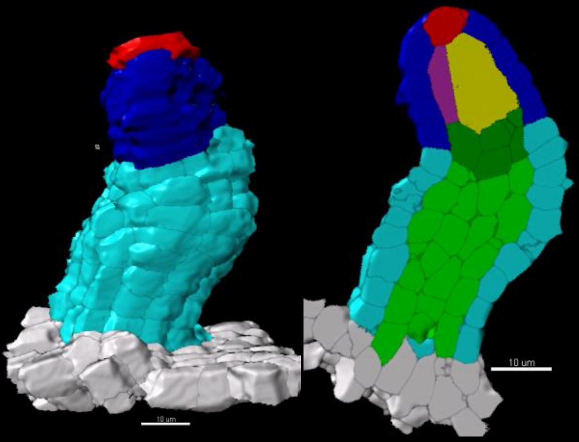

EM_C_129

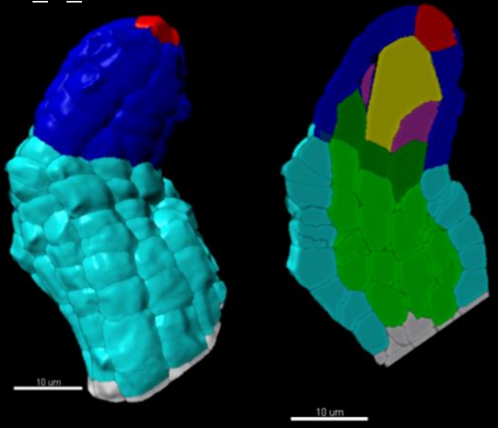

EM_C_132

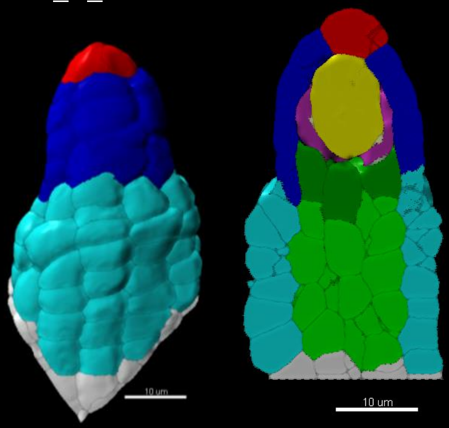

EM_C_230

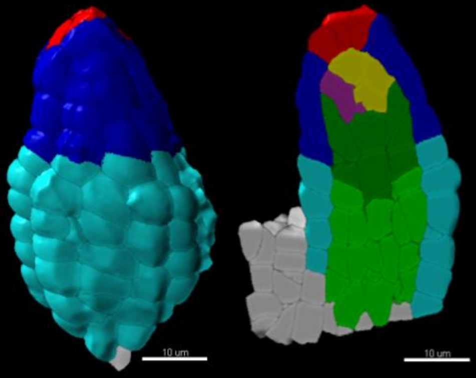

EM_C_254

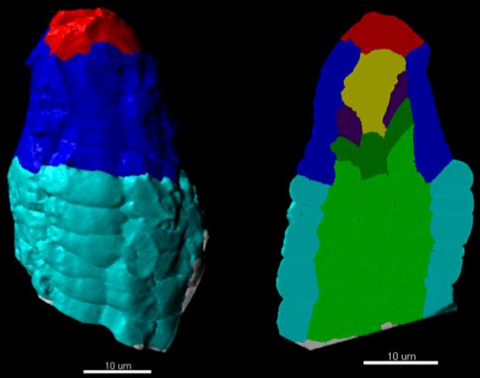

EM_C_352

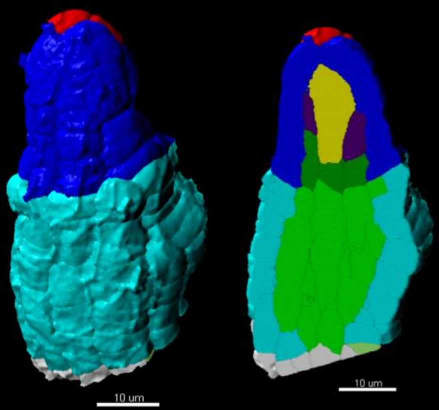

Supplement: Figure 1—source data 1. [file elife-66031-fig1-data1.pdf]
