# Supplementary material for: Organ geometry channels reproductive cell fate in the Arabidopsis ovule primordium
Source: eLife. 2021 May 7;10:e66031. doi: 10.7554/eLife.66031 (PMC8219382; doi:10.7554/eLife.66031)

Supplemental Dataset 4a- Gallery of *katanin* (*bot1-7*) and corresponding wild-type (*Ws-4*) ovules used for the 3D digital atlas related to Figure 4

Number of ovules segmented for the analysis

	bot1-7	Ws-4
Stage 0-III	n=16	n=7
Stage 1-I	n=9	n=8
Stage 1-II	n=12	n=7

bot1-7

Stage 0-III

EM_C_746

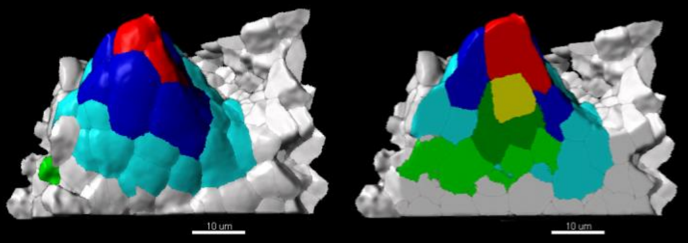

EM_C_571

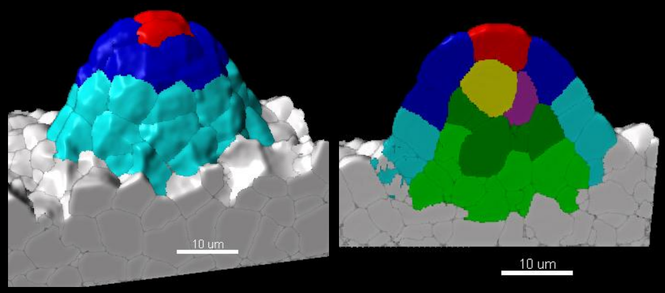

EM_C_744A

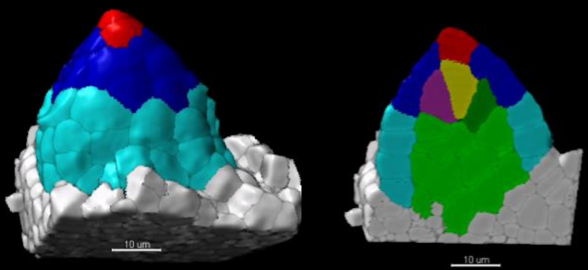

EM_C_574

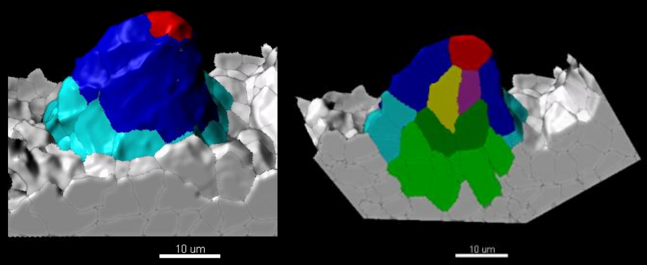

EM_C_745B

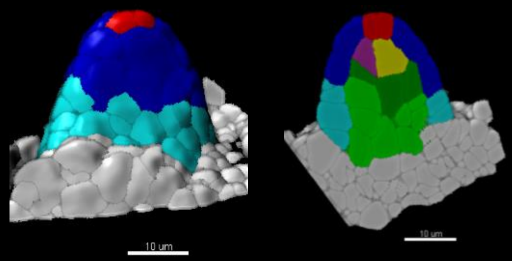

EM_C_753

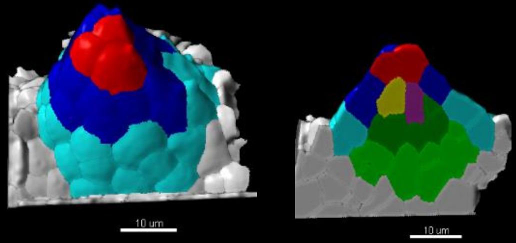

EM_C_572

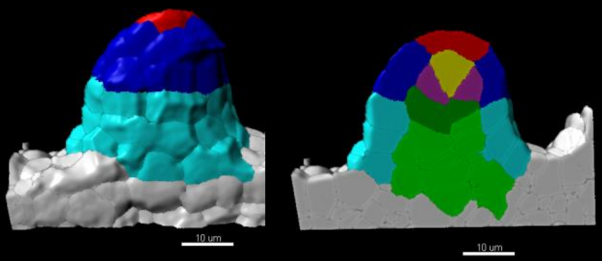

EM_C_754A

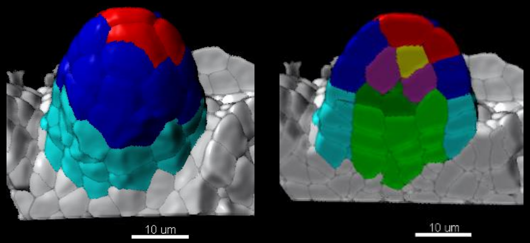

EM_C_573

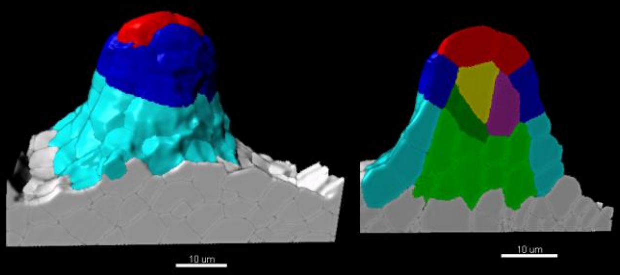

EM_C_754B

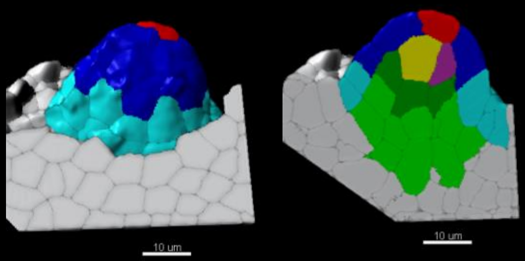

bot1-7

Stage 0-III

EM_C_280A

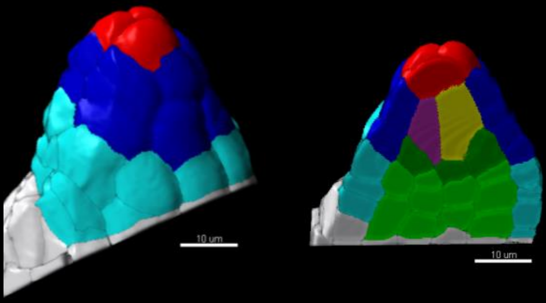

EM_C_755B

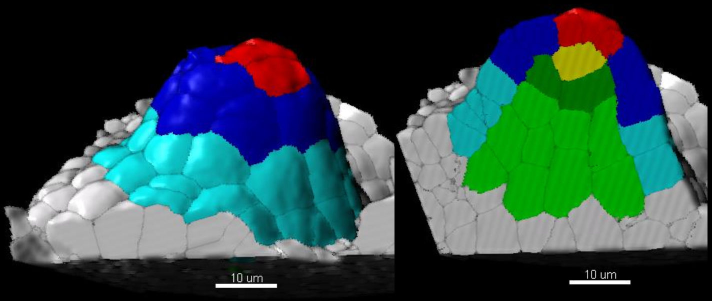

EM_C_792B

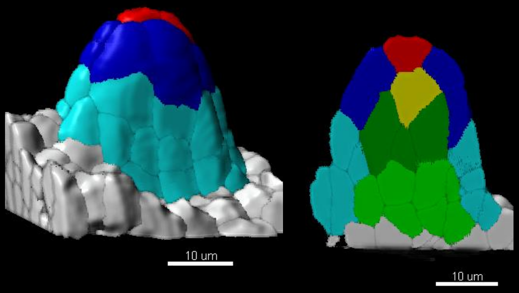

EM_C_790

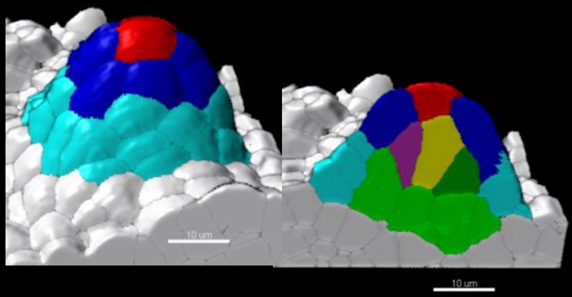

EM_C_777

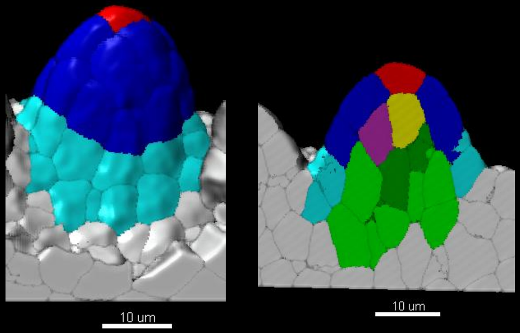

EM_C_779

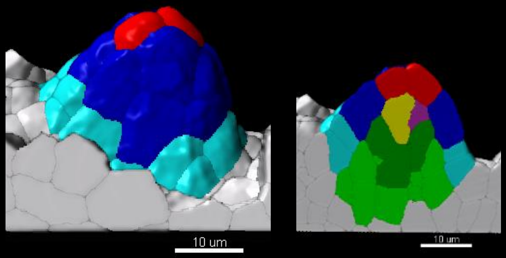

bot1-7

Stage 1-I

EM_C_743A

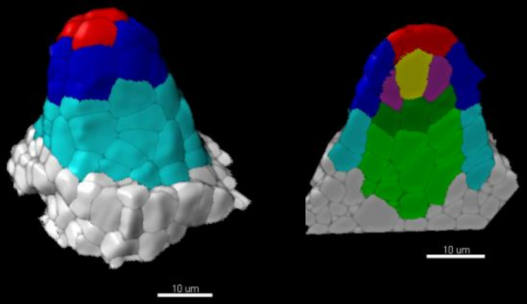

EM_C_757

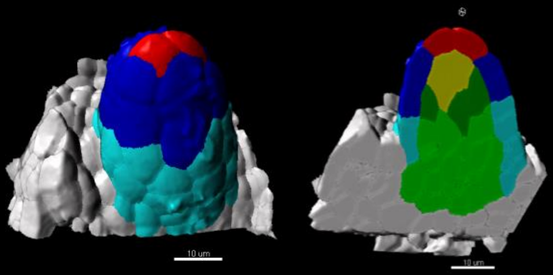

EM_C_752

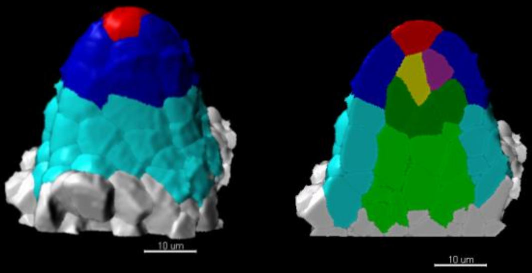

EM_C_590

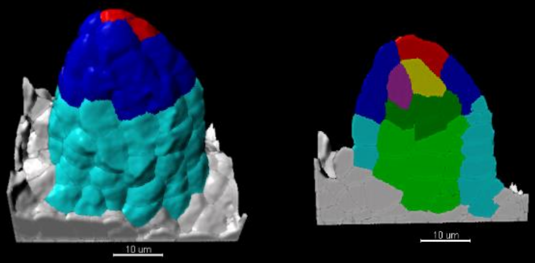

EM_C_921

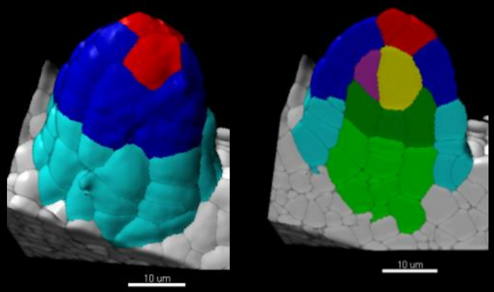

EM_C_913

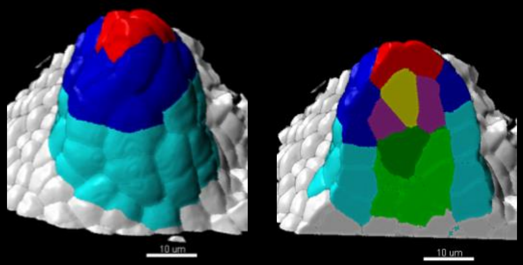

EM_C_783

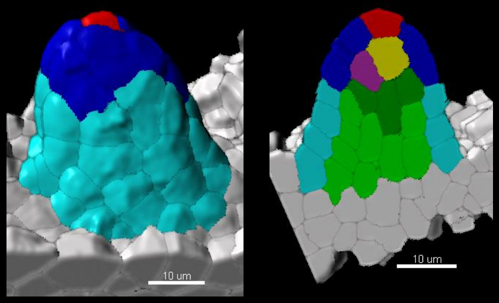

EM_C_782A

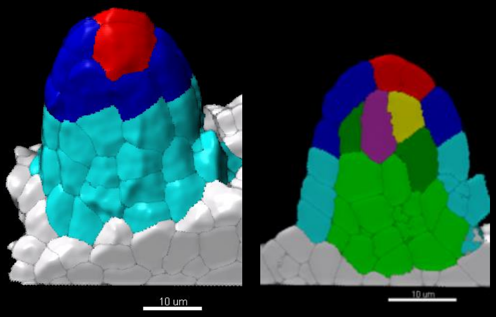

EM_C_778

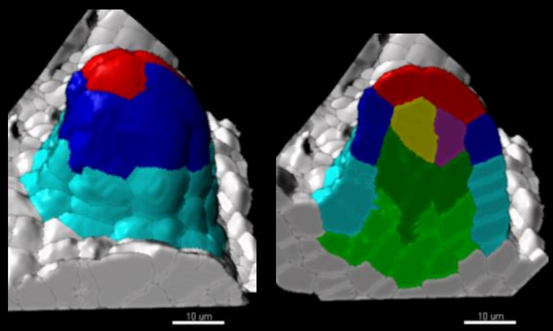

bot1-7

Stage 1-II

EM_C_914

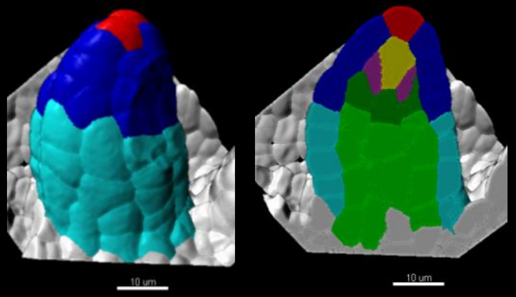

EM_C_747

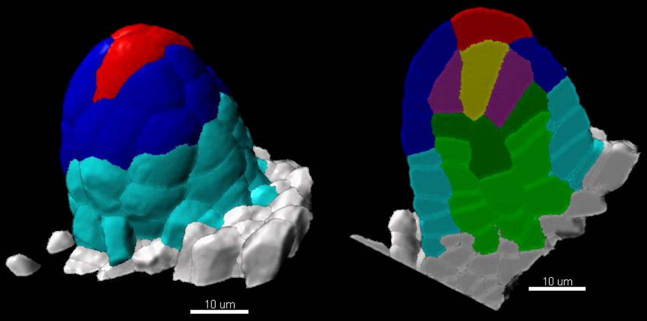

EM_C_743B

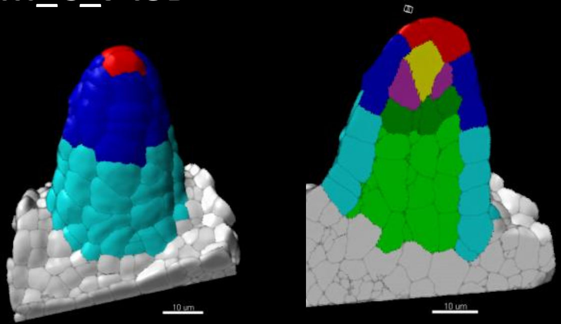

EM_C_750

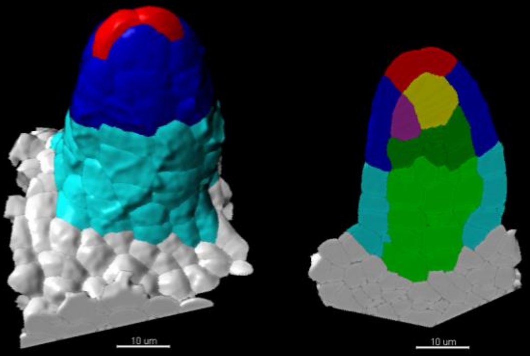

EM_C_744B

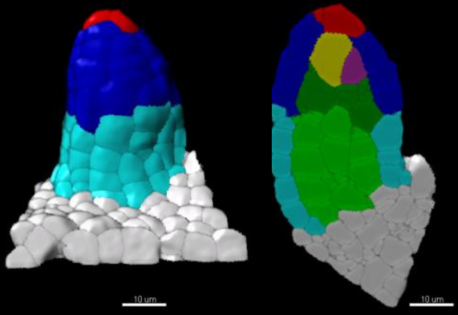

EM_C_751

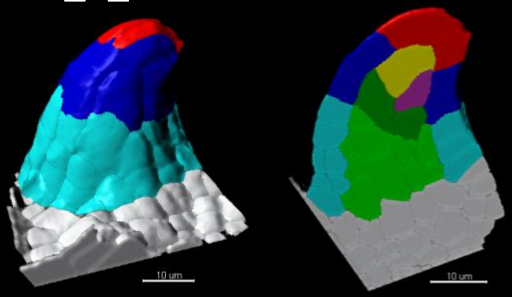

EM_C_745A

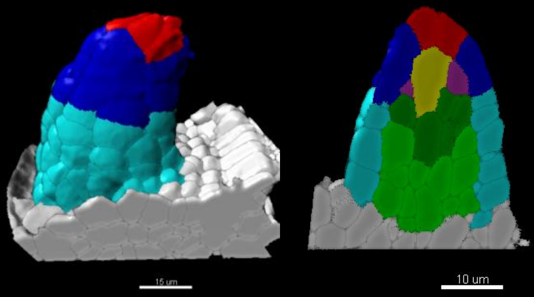

EM_C_591

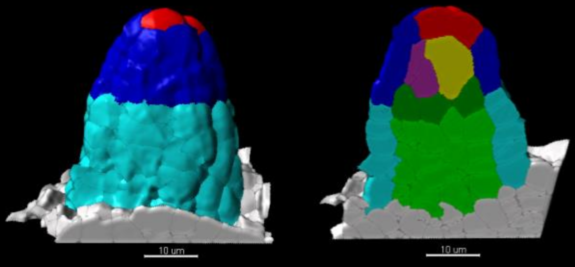

bot1-7

Stage 1-II

EM_C_755

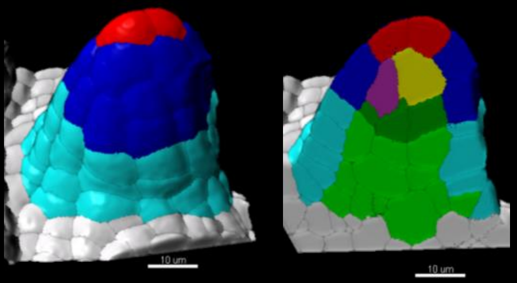

EM_C_768

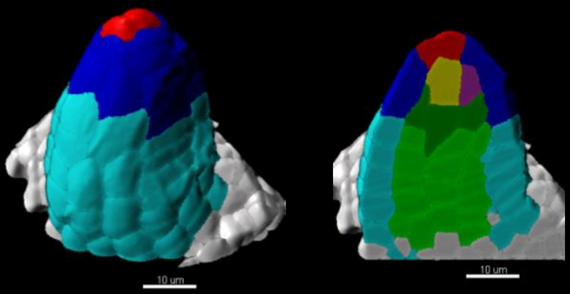

EM_C_281B

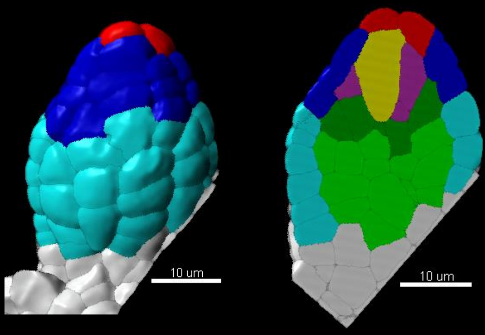

EM_C_282B

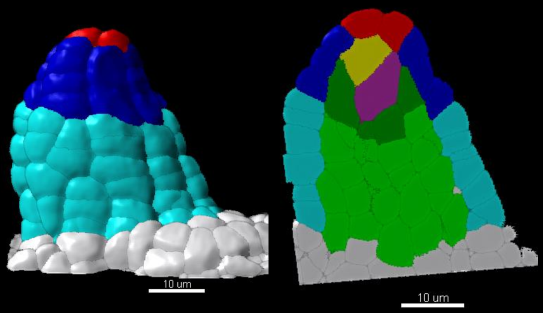

Ws4

Stage 0-III

EM_C_729B

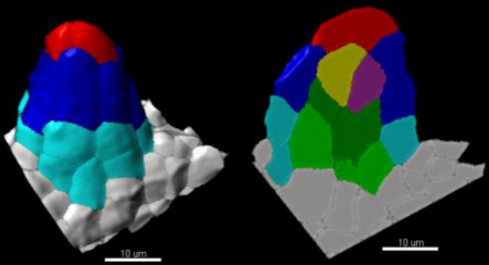

EM_C_722

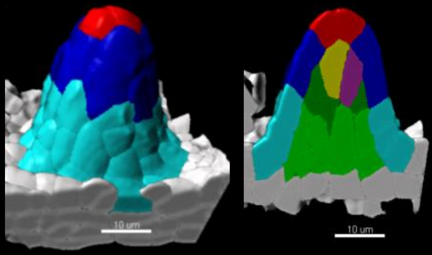

EM_C_902B

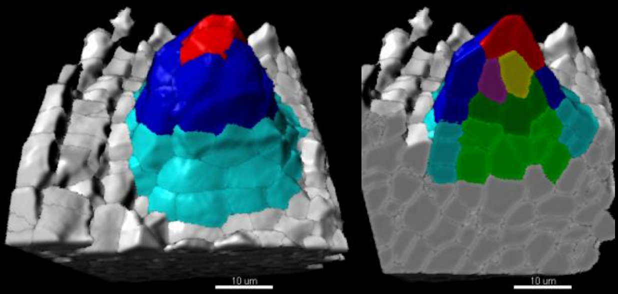

EM_C_904

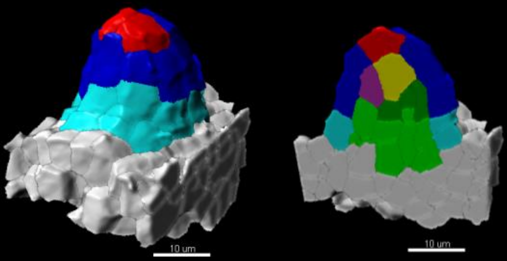

EM_C_905A

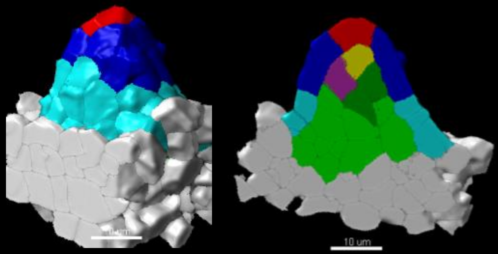

EM_C_905B

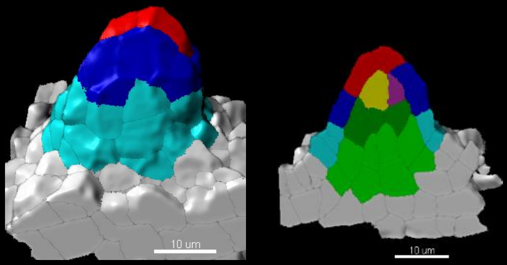

EM_C_728B

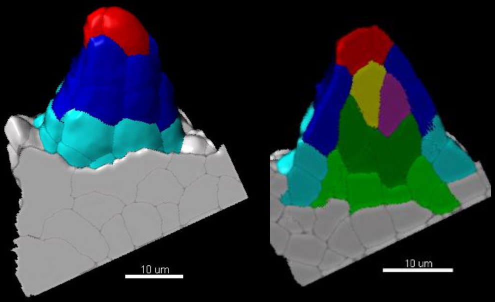

Ws4

Stage 1-I

EM_C_732

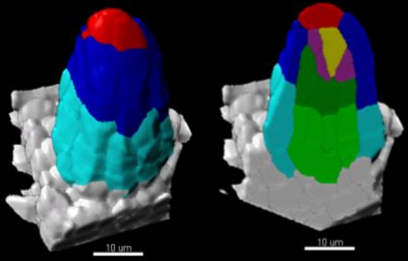

EM_C_726

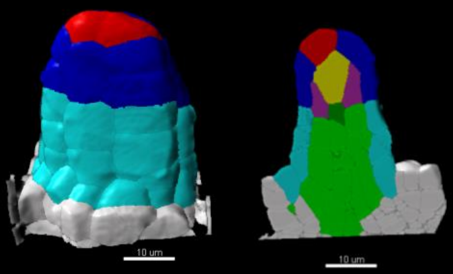

EM_C_735A

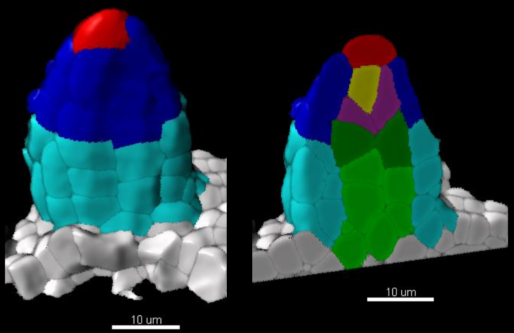

EM_C_731A

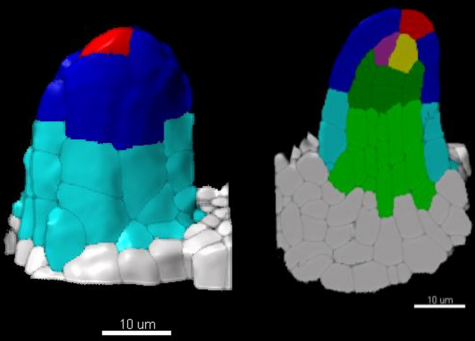

EM_C_723

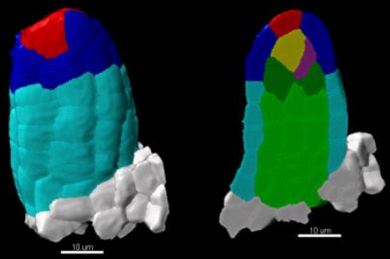

EM_C_731B

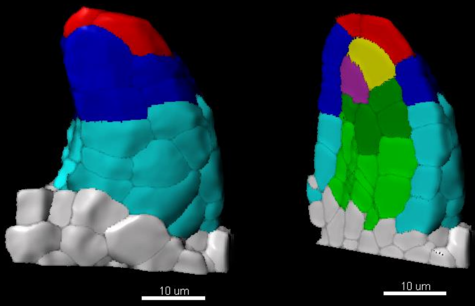

EM_C_724

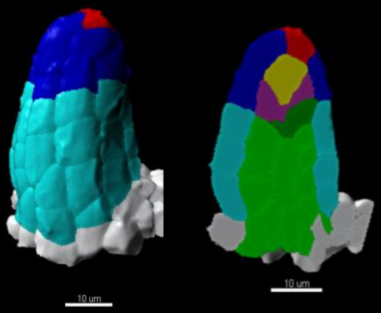

EM_C_733

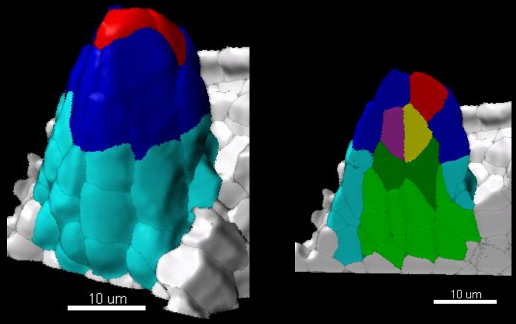

Ws4

Stage 1-II

EM_C_729A

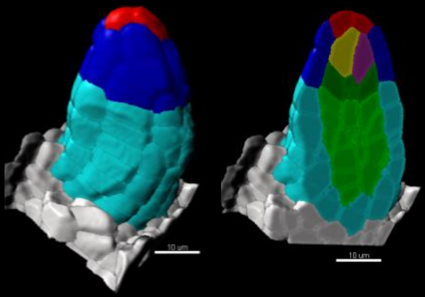

EM_C_719

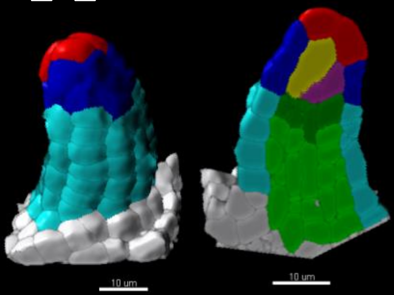

EM_C_734

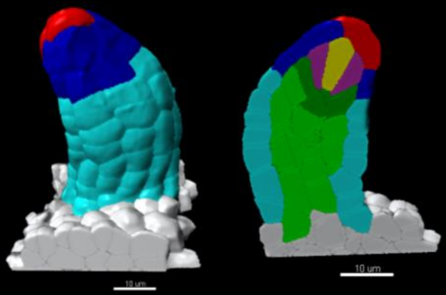

EM_C_735B

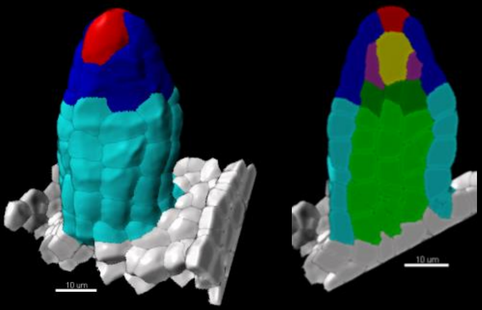

EM_C_736

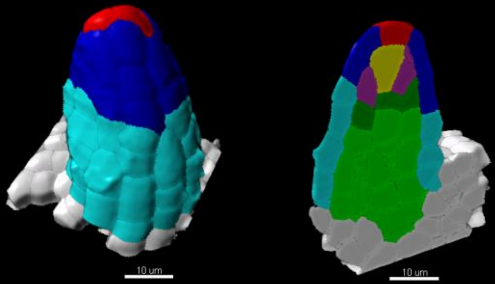

EM_C_727

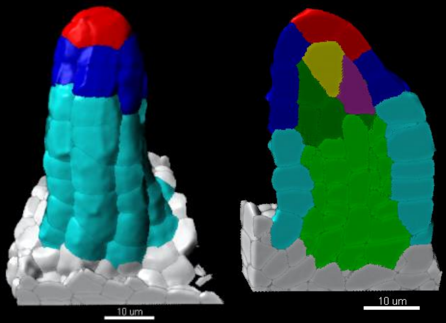

EM_C_677

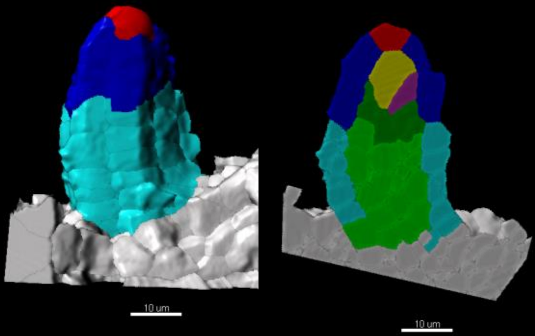

Supplement: Figure 4—source data 1. [file elife-66031-fig4-data1.pdf]
